# Influence of River Discharge on the Transport of the Saltwater Group from the North Branch in the Yangtze River Estuary

**DOI:** 10.3390/ijerph17249156

**Published:** 2020-12-08

**Authors:** Zhi Xu, Jing Ma, Hao Wang, Jianshi Zhao

**Affiliations:** 1 China Institute of Water Resources and Hydropower Research, Beijing 100038, China; xz15@mails.tsinghua.edu.cn (Z.X.); haowang1@iwhr.com (H.W.); 2 China Three Gorges Corporation, Beijing 100038, China; 3 Department of Hyraulic Engineering, Tsinghua University, Beijing 100084, China; jianshi_zhao@tsinghua.edu.cn

**Keywords:** the Yangtze river estuary, river discharge, saltwater group, MIKE21, Gompertz model, transport pattern

## Abstract

The Yangtze River Estuary (YRE) is the largest estuary in China. Recently, due to the increase of extent and frequency, saltwater intrusion has received more and more attention. In this paper, with the adoption of hydrodynamic and salinity transport mode, quantitative research of the influence of river discharge to the North Branch (NB) of the Yangtze River on the saltwater group migration law is conducted. Tide and salinity data are used to validate the model effectively. In different paths, the changes in flow and the movement of the saltwater group are similar. The saltwater group starts to move downward from the sixth day. In the staged downward movement, the larger the runoff volume, the further the distance of the core of the saltwater group, and converges to around 90 km gradually. At different flow rates, the relationship between the average location of each waterway saltwater group core tide cycle and time is consistent with the Gompertz model, and its parameters had a nonlinear relationship with the flow rate. A function is constructed to calculate the length and time of the saltwater group migration. As the flow rate increases, the faster the core of the saltwater group reaches the entrance. The downwards movement takes 3–8 days. Quantitative research on the influence of the saltwater spilling from NB to the three major reservoirs in the South Branch (SB)is conducted. The simulation results are consistent with the function calculation. River discharge has a direct impact on saltwater transport and diffusion in the YRE.

## 1. Introduction

The estuary is where the seawater and freshwater meet. Different forms of estuaries, different tides and runoff intensities will have an important impact on the mixing type of saltwater and freshwater, whereas different mixing types of saltwater and freshwater will have a significant impact on saltwater transport. Regarding large-scale saltwater intrusion, previous researchers have done a large number of research on the length of saltwater intrusion. Earlier studies of saltwater intrusion length assumed that runoff and tidal transport of saltwater were balanced [1,2], and on this basis, many researchers proposed estuarine salinity transport balance equations. Festa and Hansen [3] used a two-dimensional mathematical model to discover that the length of salinity intrusion would change with the estuary water depth, vertical diffusion coefficient and runoff. Chatwin [4] further provided the salinity intrusion length, including estuary depth, runoff, vertical diffusion coefficient, estuary salinity, etc.

With the development of computer technology, more and more researchers begin to use mathematical models to study the saltwater intrusion. Park and Kuo [5] used a two-dimensional mathematical model to derive the response process of the length of saltwater intrusion to the periodic variation of large and small tides. Banas et al. [6] pointed out that the estuary’s response to external changes depends on the estuary response time scale and the external time scale. Nebiyu [7] used a three-dimensional numerical model to study the process of saltwater intrusion in the estuary under climate change. Meselhe and Noshi [8] used the finite difference method to construct a three-dimensional numerical model at the Calcasieu-Sabine estuary, and simulated the hydrodynamic and salinity distribution of the estuary. Kurup et al. [9] applies a two-dimensional numerical model to the Swan estuary, and analyzed and calculated the impact of different sea inflows on the location and extent of saltwater wedges in different seasons in the area. Acertsl [10] and Nguyen [11,12] et al. used this model to analyze and study the saltwater intrusion in the Gorai and Mekong estuaries, respectively. Tiruneh, N.D. et al. [7] used a three-dimensional numerical model of the estuary to simulate and analyze the effects of changes in water volume and sea level on estuarine saltwater intrusion. Bhuiyan and Dutta [13] reported a 1 m sea level rise (SLR) could produce an increase of 1.5 psu in the salinity in the Gorai river network, Bangladesh. A salinity model study due to SLR in the James River (USA) showed that salinity could intrude about 10 km farther upstream for a 1 m SLR [14]. Grabemann et al. [15] reported a 2 km upstream advance of the brackish water zone in the approximately 80 km long Weser Estuary in Germany for a 0.55 m SLR scenario. In the Chesapeake Bay, the mean salinity, salinity intrusion distance and stratification would increase with rising sea level [16].

Saltwater intrusion occasionally occurs in the Yangtze River Delta and the Pearl River Delta during the dry season in China. At the same time, the estuary is usually one of the most densely populated and economically developed regions. With the development of economy, the demand for freshwater for industry, agriculture and urban living is increasing. The estuary is the most direct and important source of freshwater, and the intrusion of saltwater in the estuary is the main obstacle affecting the utilization of freshwater resources in this area [17]. The saltwater intrusion not only has a negative impact on the population of the estuary, but also affects the hydrological environment. The saltwater intrusion causes huge losses to various countries every year [18,19,20].

As the largest estuary in China, the Yangtze River estuary has always been the focus of saltwater intrusion research. The studies began in the 1980s. In 1980, Shen Huanting [21] conducted a preliminary study on saltwater intrusion in the Yangtze River estuary, provided the distribution characteristics of saltwater in the Yangtze River estuary and mentioned the important phenomenon of spilling from the North Branch (NB) of the Yangtze River estuary. The Yangtze River estuary belongs to the middle tidal estuary, which is dominated by half-day tides, and its upstream runoff is abundant. The interaction between tide and runoff has become the emphasis of many scholars’ research, including Mao Zhichang et al. [22], Xiao Chengtai et al. [23], Song Zhirao et al. [24], Jianrong Zhu et al. [25] and Yazhen Kong et al. [26]. With the development of computer technology, scholars have studied the Yangtze River estuary saltwater intrusion through numerical simulation quantization. Wu Hui [27] used the ECOM-si model to study the quantitative relationship between saltwater spillover, runoff and tidal range in the NB of the Yangtze River estuary. Chen Li [28] used the statistical law to establish a saltwater intrusion prediction model for Chenkeng reservoir in the Yangtze River estuary. Xu Zhi et al. [18] used the MIKE21 model to study the relationship between river discharge of the Yangtze River estuary and saltwater intrusion, and initially established a saltwater intrusion function. Li Lu [29] pointed out that Stokes transportation was the main reason for NB spillover, and the tidal pump was the main reason for NB saltwater spillover. Qiu Chen et al. [30] did quantitative research on the changes of saltwater intrusion characteristics in the Yangtze River estuary after sea level rises in the future.

Regarding the study of saltwater spillover in the NB of the Yangtze River estuary, it is difficult to observe the path of NB spillover visually and determine the salinity distribution of the NB spillover in some areas (Qingcaosha, Beigang and Nangang), due to the mutual mixing and doping of the saltwater intrusion from the South Branch (SB) and the spillover saltwater from the NB. In the past, researchers mainly depended on the relationship between the peak and the valley values of the salinity and the tide pattern, the phase relationship between the salinity process and the flow velocity, and the vertical distribution of the salinity to determine the main source of the saltwater [31]. Some scholars have qualitatively divided the estuary area of the NB spillover and the conventional offshore on the basis of the changes in salinity values [32]. There is a lack of clear and accurate methods to reflect the time varying process of the NB spillover under various conditions, and the specific distribution of spillover saltwater in the SB. In addition, the topography of the NB has changed significantly in recent years, and there is still no relevant research on its influence on the saltwater spillover on the NB of the Yangtze River estuary. The advantages of the MIKE21 model include the following: (1) an unstructured mesh with a high resolution was applied, which better fit the shoreline areas; (2) the influences of Hangzhou Bay were considered and (3) the use of spatially varying bottom roughness was included in the experimental process. This paper combines the measured data, builds a MIKE21 model, which simulates the movement of spillover saltwater group and salinity variation, under the influence of NB of Yangtze River estuary alone, and discusses the effects of factors, such as runoff, tidal current and wind on the activity and concentration of the saltwater group.

## 2. Materials and Methods

### 2.1. Study Site

**Figure 1:**
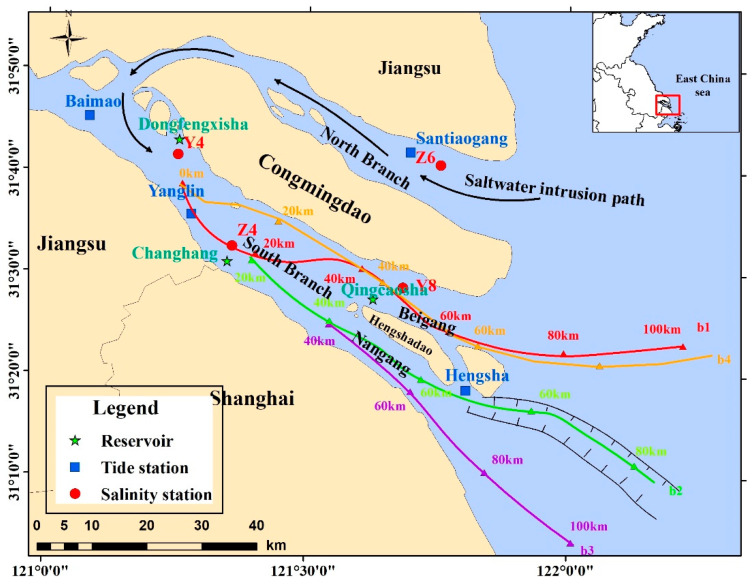
The range of the Yangtze Estuary.

The Yangtze River Estuary is China’s most populated and industrially productive area. The country’s three major drinking-water reservoirs (Qingcaosha, Chenhang and Dongfengxisha Reservoirs) are located in the Yangtze River Estuary (YRE) (Figure 1). These reservoirs provide abundant water sources for the cities in the estuary area, including those of Jiangsu Province and Shanghai Municipality.

The Dongfengxisha Reservoir is located on the north side of the upstream of SB, with a total capacity of up to 9.762 million m^3^. The Chenhang Reservoir is located in the downstream area of Lihekou and has a comparatively small capacity. The Qingcaosha Reservoir, which is located in the northern part of Changxing Island, has an area of 70 km^2^, and a capacity of 0.44 billion m^3^. The water quality in the Qingcaosha reservoir is considered to be very high (Category I or II) according to Chinese surface water standard, which makes the Qingcaosha Reservoir one of the most efficient reservoirs in China at this time.

The transport route of the NB of the Yangtze River estuary to the SB is divided into four paths, b1, b2, b3 and b4 (Figure 1). The route division is based on the topography of the Yangtze River Estuary and previous research results. The original point of the path is 40 km away from the sandy tail of Baimaosha to the entrance. Path b is divided into b1, b2, b3 and b4 in the horizontal direction (Figure 1), where path b1 is the main waterway of the SB to Beigang, which is about 100 km; path b2 is the main waterway of the SB to Nangang to Beicao, which is around 100 km; path b3 is the main waterway of SB to Nangang to Nancao, which is around 100 km; path b4 enters the Xinqiao waterway to Beigang from the main waterway of SB along the upper channel of the Xinqiao waterway.

### 2.2. Model Creation

MIKE 21 was used to create the hydrological model. Details of MIKE21, and the model creation are given in Xu et al. [17].

**Figure 2:**
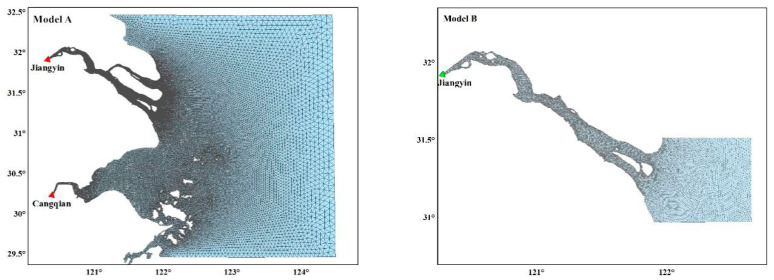
The mesh of Model A and B.

In order to facilitate the study of NB spillover, Models A and B are built (Figure 2). The range of Model A is the largest. The upstream reaches Jiangyin station, and the downstream offshore boundaries are taken from the south, north and east open boundary, respectively. The northern boundary is near Lusi port, the southern boundary reaches the south of Zhoushan island, and the location from east boundary to open sea where the depth is 50 m. Model B is created especially for the study of the activity of the saltwater group in the NB of the Yangtze River separately. Its upstream boundary is to Jiangyin and downstream NB boundary is set near Lianxing port. The east boundary of the SB is about 100 km away, 40 m depth from the entrance. Its north boundary is near the north of Chongming East beach, and the south boundary is near Luchao port. The computational domain of Model A was composed of an unstructured triangular mesh with 54,243 nodes and 92,771 elements. The meshes of Model B were 32,311 nodes and 60,368 elements.

The land boundary of the model required the conditions of “non-importable and slippery”. That is to say, the flow velocity was zero along the normal direction of the closed boundary. In the open sea areas, a tidal wave model was used to calculate the tide levels of the three outer boundaries of the outer sea. In this study, the global tidal wave model TPXO6.2 [33] was selected. The open boundary varied with time. The initial tidal level and velocity are defined as 1 m and 0 m/s, respectively.

According to the flow correlation analysis between Datong and Jiangyin station [34]. The runoff processes of the two stations were found to be similar. Therefore, the discharge data of the Datong Station was selected as the flow boundary of Jiangyin. The flow boundary of Cangqian was determined to be 1000 m^3^/s. It is an average flow, the impact on the model can be ignored. During the experimental process, dry and wet grids were used to discriminate the model shoals. The values of hdry = 0.005 m, hflood = 0.05 m and hwet = 0.1 m were utilized for each parameter [35].

The upstream boundary salinity for Model A was set to 0, and the salinity at the southern boundary was obtained through linear interpolation between 15‰ to the west and 30‰ to the east. The salinity at the eastern boundary was obtained through linear interpolation between 30 to the south and 35‰ to the north. The salinity of the northern boundary was obtained through linear interpolation between 25‰ to the west and 35‰ to the east. The salinity at the boundary of Model B in the North Branch was extracted from salinity calculation results of Model A, and the salinity at the boundary in the southern branch was set to 0.

### 2.3. Model Calibration and Validation

**Figure 3:**
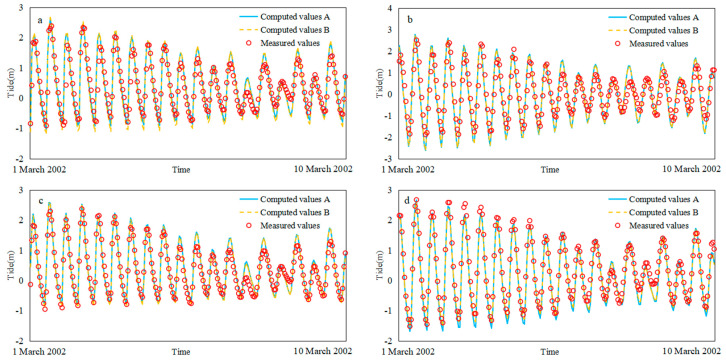
Verification of tidal level for Models A and B: (**a**) Baimao, (**b**) Santiaogang, (**c**) Yanglin, (**d**) Hengsha.

**Figure 4:**
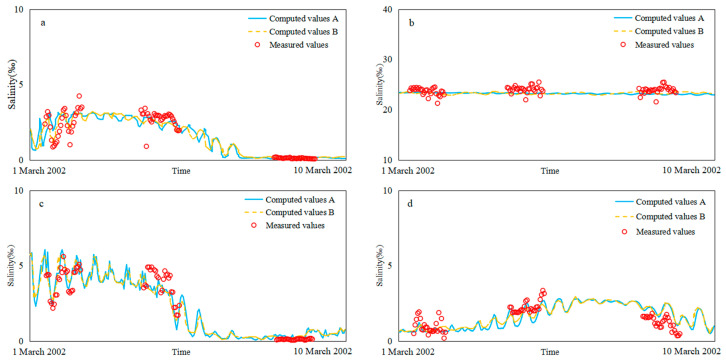
Verification of salinity level for Models A and B: (**a**) Z4, (**b**) Z6, (**c**) Y4, (**d**) Y8.

Hydrodynamic verification includes tide level verification and salinity verification. Four tide level stations (Baimao (Bm), Santiaogang (Stg), Yanglin (Yl) and Hengsha (Hs)) and four salinity stations (Z4, Z6, Y4 and Y8) were verified. The salinity was measured as exploration, and the Knudsen salinity formula [36] was used for the conversion. Validation time: from 00:00 on 1 March 2002 to 00:00 on 11 March 2002. The tidal level validation and salinity validation of Models A and B are shown in Figure 3 and Figure 4.

The skill model [37] was used to test the effect of Mike 21 model, the expression is as follows:

(1) $$skill=1-\frac{\Sigma_{i=1}^{N}|M-D{|}^{2}}{\Sigma_{i=1}^{N}{\left(|M-\overset{-}{D}|+|D-\overset{-}{D}|\right)}^{2}}$$

where *M* is the calculated value; *X* is the measured value and *N* is the number of data. A skill value of 1 indicates that the simulation effect is identical, 0.65–1 indicates excellent, 0.5–0.65 indicates very well, 0.2–0.5 indicates well and less than 0.2 indicates not well.

Model-observation data comparison statistics for tidal and salinity is shown in Table 1. It can be seen that all stations skill values were greater than 0.65, which was excellent. The tide values were above 0.9, and the salinity values ranged from 0.70 to 0.82. The direction and intensity of seawater flow affected the diffusion of salinity. Some of the upstream salinity stations had low salinity and were very sensitive to water flow. There will be some deviation during the simulation.

## 3. Results

### 3.1. Calculation Results in Different River Discharges

#### 3.1.1. The Position Change of the Saltwater Group

**Figure 5:**
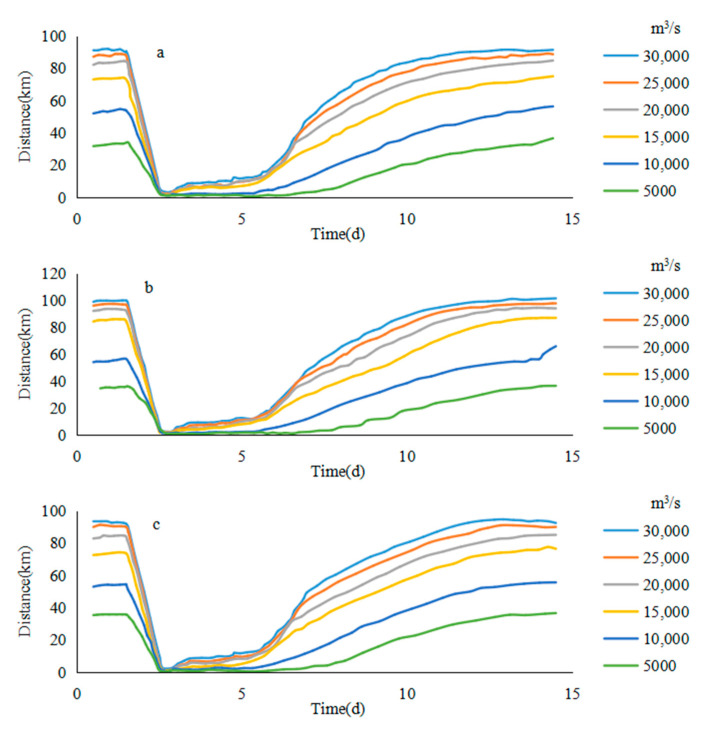

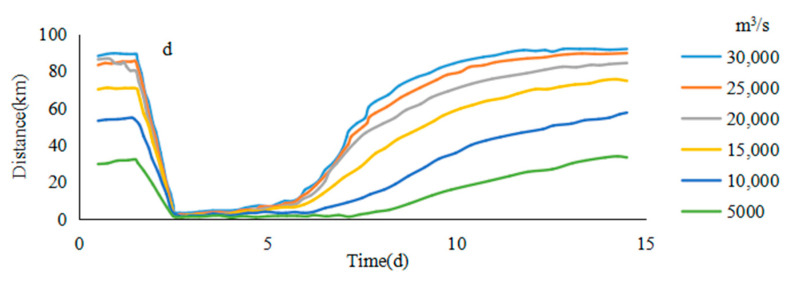
Average location of the saltwater group in different river discharges: (**a**) b1, (**b**) b2, (**c**) b3, (**d**) b4.

In this paper, the maximum value of saltwater group on the path was used to represent the core of saltwater group. The calculation length of each flow value at the upstream of Jiangyin was always consistent with the control mode, and both were one month. The average annual river discharge of the Yangtze River estuary was 27,856 m^3^/s, and the average river discharge in the dry season was 16,543 m^3^/s, sometimes as low as 10,000 m^3^/s. In order to describe the effect of flow interval change on salinity. The flow increased from 5000 to 30,000 m^3^/s, and each additional flow was 5000 m^3^/s. There were six different flow values. The model operation results are shown in Figure 5.

It can be seen from Figure 5 that on different paths, the rules of the downward movement of the saltwater group core under various flow conditions were basically similar. Taking b1 as an example, it can be seen from the moving position of the saltwater group core on the path b1 under different runoff conditions that before the sixth day, since the spillover was relatively stable, the saltwater group of high concentration brine was near the original point and did not move too much. Until six days later, the tide difference in Qinglong port weakened significantly comparing to the previous tides, and spillover at the NB declined to a certain extent, causing the saltwater group core to start to move downwards from the sixth day.

**Figure 6:**
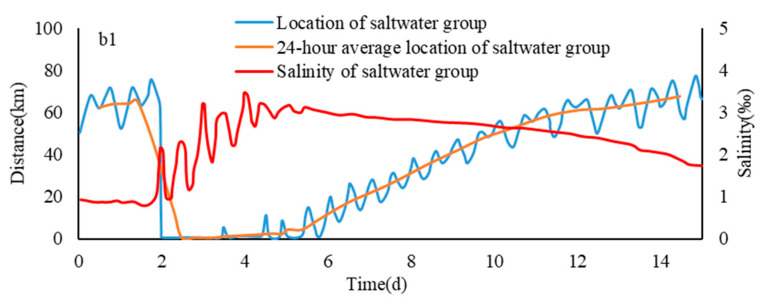
The schematic figure of the saltwater group transport.

From the sixth day, the downwards movement of the initial saltwater group got slower. As the upstream spillover saltwater dropped sharply, the upstream saltwater supply was cut off by the drained runoff and the movement of the core of the saltwater group accelerated gradually. The larger the runoff value, the faster the core of the saltwater group moved down during this period. As the position of the saltwater group moved down, the advantage of the downward flow diminutions gradually increased, especially after the entrance (after 60 km). Due to the tidal support effect, the downward movement speed of the saltwater core weakened and stopped gradually. At the end of the downward movement, the larger the runoff value, the further downward movement reached by the saltwater core, and converged to around 90 km gradually. The schematic figure of the saltwater group transport is shown in Figure 6.

The cores of the saltwater group on the other paths were basically similar to the b1 rule under different flow conditions. More specifically, since b2 passed through the deepwater channel project, due to its influence, the maximum displacement of the core of the saltwater group was slightly larger than other paths. Furthermore, the speed of moving downwards of the saltwater group core had a significant increase in the process of entering the deepwater channel.

#### 3.1.2. Salinity Change of the Saltwater Group

**Figure 7:**
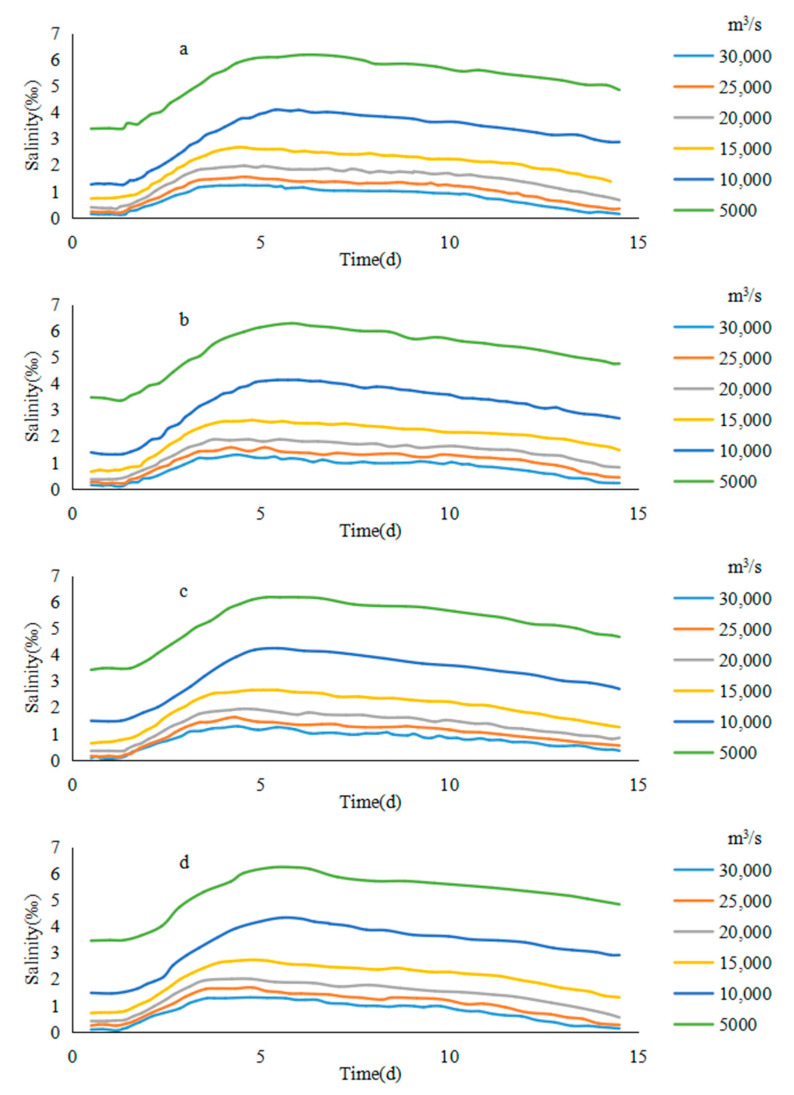
The average salinity of the saltwater group in different river discharges: (**a**) b1, (**b**) b2, (**c**) b3, (**d**) b4.

On different paths, the average salinity trends of the core of the saltwater group were similar under different flow conditions. Taking the path b1 as an example, from the 24 h average salinity of the saltwater group core on path b1 under different runoff conditions, it can be found that the concentration of the saltwater group core increased significantly during the 3–6 days of the high tide period. As the tidal range became small and the spillover weakened, the saltwater group moved downward from the sixth day. During the downward movement, due to the tidal dispersion and turbulent diffusion, the concentration of the saltwater group core began to decrease (Figure 7).

In the case of the low flow rate (Q < 10,000 m^3^/s), the decreasing speed of the salinity of saltwater core was almost uniform due to the close downward movement distance of the saltwater group within the entrance. In the case of a high flow rate (Q > 10,000 m^3^/s), as the core of the saltwater group will move beyond the exit in the later period (after 60 km), the lateral diffusion of the saltwater group was intensified, and the concentration decrease rate of the saltwater group increased significantly, because it was not constrained by the land boundary. In this way, the inflection point of the concentration course line of the saltwater group core appeared during the descent. As the flow rate was high, the earlier the core of the saltwater group entered the open sea, the sooner the inflection pint that marked the accelerated decline in salinity would appear.

On the other paths, the salinity of the saltwater group core was basically similar to the b1 rule under different conditions. On the path of b2, due to the restrictions of double jetties, under the condition of Q = 15,000 m^3^/s, the salinity decay rate of the saltwater group core did not increase significantly. However, with the increase of the flow, the downward movement distance of the saltwater group core increased, the core of the saltwater group increased gradually under the influence of tidal turbulent diffusion response in the open sea, and the decay rate of the saltwater group core increased gradually.

### 3.2. Analysis of Transport Law of the Saltwater Group

#### 3.2.1. Fitting Transport Position of the Saltwater Group in Different River Discharges by Curve Function

As can be seen from the above section, there are great differences in the speed and distance of the saltwater moving downwards under different flow conditions. As the flow rate increased, the speed and distance of the saltwater group core descent increased. However, the overall rule of the downward movement of the saltwater group was basically similar.

In order to better describe the above mentioned downward movement law of the saltwater group core, the Gompertz model [38] was used here to fit the movement position of the core of the saltwater group on each path under different runoff conditions. Since the calculation of the downward movement of the saltwater group core started at 0:00 on the sixth day, the expression is as follows:

(2) $$y=a\exp(-be^{-c(t-5)})$$

where, *t* stands for time; *a, b* and *c* all are parameters fitted out by the Gompertz model based on numerical simulation (*a* represents the limit value of the curve, and is the maximum displacement of the saltwater group theoretically, *b* mainly represents the initial value relative to the limit value and *c* represents the discharging rate of the saltwater group). The bigger the *c* is, the faster the saltwater group discharge, and more quickly, the saltwater group can reach the maximum theoretical displacement of the saltwater group.

Equation (2) is based on the results of the numerical simulation. The changes in the position of the saltwater group core at different levels of flow on the path b1 are fitted according to the principle of the least square [39]. The parameters of the fitting curve of the Gompertz model are shown in the Table 2 below.

It can be found in the table that there is a positive correlation between parameter value a and runoff value, and converge to about 90 gradually, indicating that the larger the runoff is, the larger the theoretical maximum displacement reached by the core of the saltwater group. The maximum displacement was about 90 km. In addition, it is worth noting that when the runoff was low (Q < 10,000 m^3^/s), the theoretical maximum displacement was within 60 km, illustrating that under the condition of low runoff, the saltwater group core cannot enter the open sea through the SB during the half-moon cycle. The saltwater spillover in the first half of the month overlaps with the one in the second half of the month, which causes severe saline pollution. It is consistent with the results of the study by Xiao Chengyou et al. [40].

It can be seen from the table that the value of *b* decreased first and then increased with runoff. The value of *c* also decreased first and then increased with the runoff. This is mainly because, in the early stage of the runoff increase, the theoretical maximum displacement of the core of the saltwater group will greatly increase with the increase of the runoff due to the small base number of the runoff, which will lead to a significant increase in the time to reach the theoretical maximum displacement, so that the value of *c* decreases. With the further increase of the runoff, *t*, the position of saltwater mass is approaching to 90 km, and the *c* increases with the increase of runoff.

**Figure 8:**
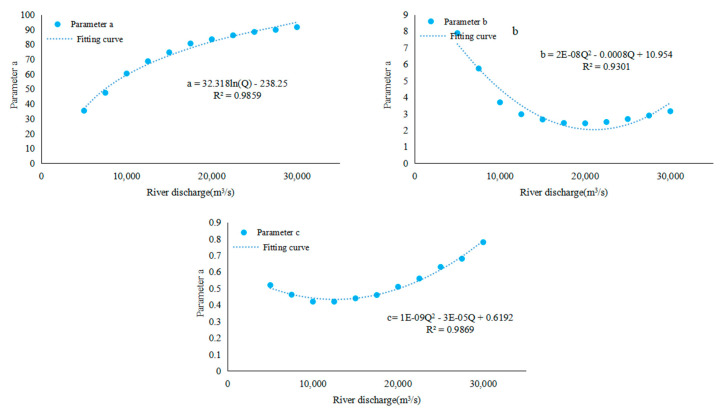
The relationship between different parameters and river discharges: (**a**) parameter a, (**b**) parameter b, (**c**) parameter c.

In order to combine the flow rate Q with the downward Equation (2), the following functions a, b, c and Q are fitted (Figure 8) as follows:

(3) a=32.318ln(Q)−238.25

(4) $$b=2\times 10^{-8}Q^{2}-0.0008Q+10.954$$

(5) $$c=1\times 10^{-9}Q^{2}-3\times 10^{-5}Q+0.6192$$

In this way, the value of parameters *a, b* and *c* can be directly calculated based on the flow rate Q, and then position of the saltwater group core under different flow conditions can be calculated (Figure 8) according to Equation (2). From the perspective of the fitting effect, it establishes the relationship between the downward movement of the saltwater group core and the Q, t function. It reflects the relationship between the downward movement velocity of the saltwater group and Q, which is more generalized.

**Figure 9:**
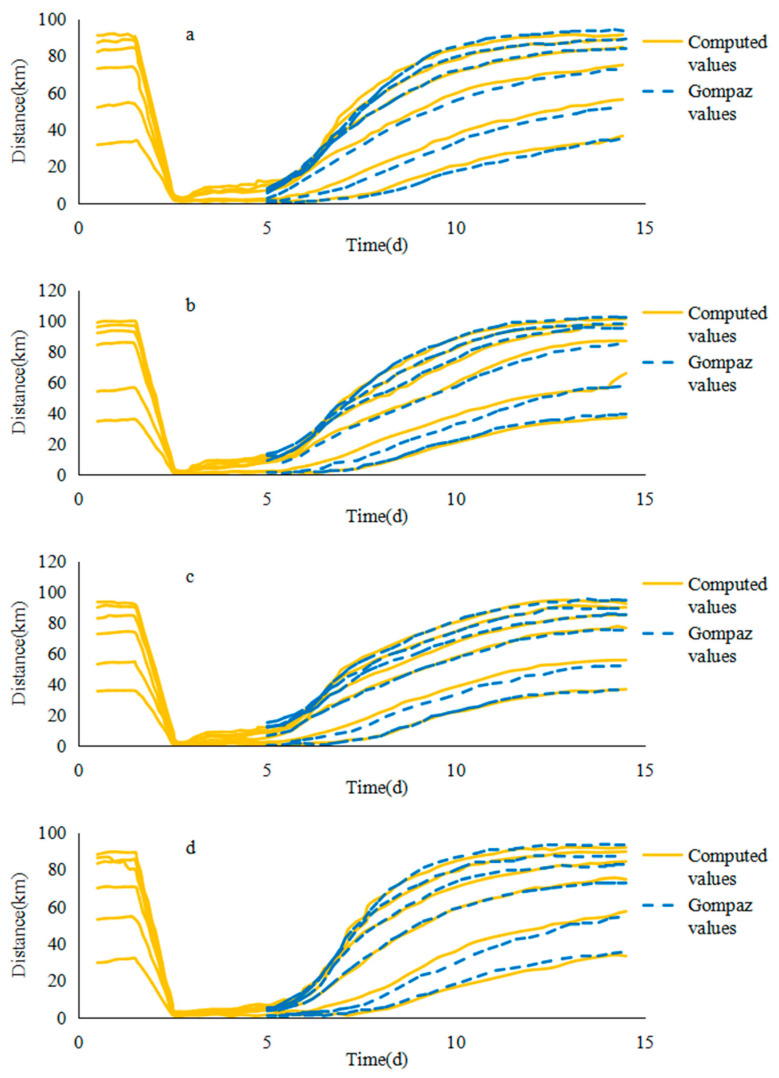
Fitting curve of different routes: (**a**) b1, (**b**) b2, (**c**) b3, (**d**) b4.

With reference to the research method of the movement of the saltwater group core in the path b1, the relationship between the relevant parameters of b2, b3 and b4 Gompertz model curves and the flow rate was calculated. Sequentially, a process line of 24 h average position of the saltwater group core under different runoff conditions on different paths variation with time was fitted (Figure 9).

#### 3.2.2. The Time When Saltwater Group Reaches the Entrance in Different River Discharges

In the actual production and scientific research process, people always draw more attention to when the saltwater group moves down to a certain position. Since the relationship between the parameters a, b, c and the flow was obtained above, *y* in the formula can be set to a certain value according to the needs of the research site (that is the distance between research point and saltwater group original point). In this way, it is possible to calculate when the saltwater group core reaches a certain point under a certain flow. Only the time interval required for the saltwater group reaching the entrance (*y* = 60 km) from the original point under different flow conditions on different paths was calculated here. The formula is as follows:

(6) t=−1/c*ln[−1/bln(y/a)]+5

**Figure 10:**
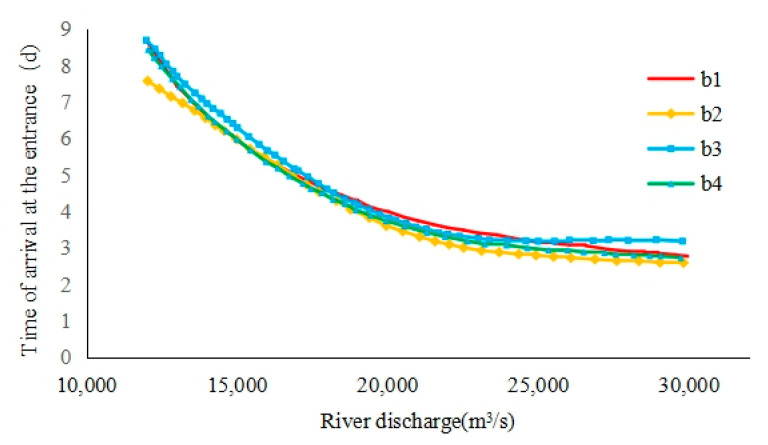
Achieving the entrance time of the saltwater group in different conditions.

According to Table 3 and the formulas, the time required for the saltwater group to reach the entrance at different flow rates can be obtained, as shown in Table 4 and Figure 10. It could be found that with the increase of runoff, the time for the saltwater group core to reach the entrance on each path became shorter. Under the flow condition of 10,000 m^3^/s, it took 7–8 days for the saltwater group core to reach the entrance. At a flow rate of 30,000 m^3^/s, it only took three days for the saltwater group core to reach the entrance on each path.

On different paths, it took a slightly longer for the core of the saltwater group in path b4 than path b1 to reach the entrance. This was mainly because the b4 path passed through the rising tide water channel on the northern shore of the SB, which caused the slowdown of the saltwater core’s downward speed. The time required for the saltwater group on the path b2 to reach the entrance was shorter than that of b3. This is mainly due to the effect of deepwater channel construction, the drainage advantage of the water body increased, which made the saltwater group core move down faster.

### 3.3. Relative Salinity Change of the Saltwater Group in Different River Discharges

In order to further understand the relationship between the salinity of the saltwater group and the runoff during the downward movement, it is necessary to focus on the changes of the concentration of the saltwater group core after the sixth day.

Figure 6 demonstrates the salinity change of the absolute value of the saltwater core, but in the process of the substance concentration changing, research often pay more attention to the relative change of the substance concentration [41], that is, the change rate of the substance concentration relative to the initial concentration, for the purpose of investigating the relative decay rate of the salinity of the saltwater group core.

**Figure 11:**
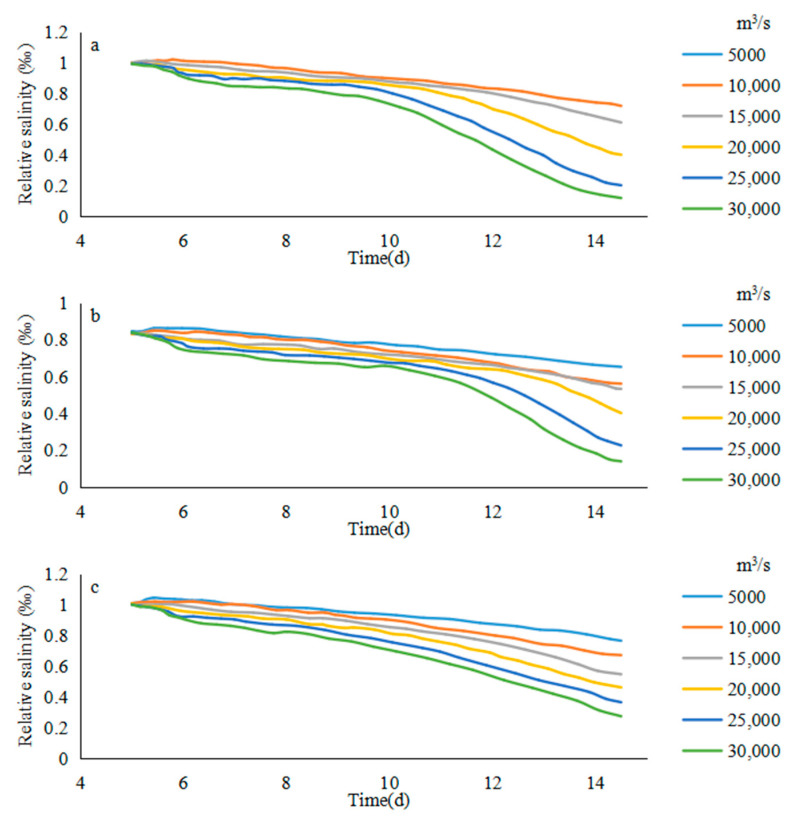

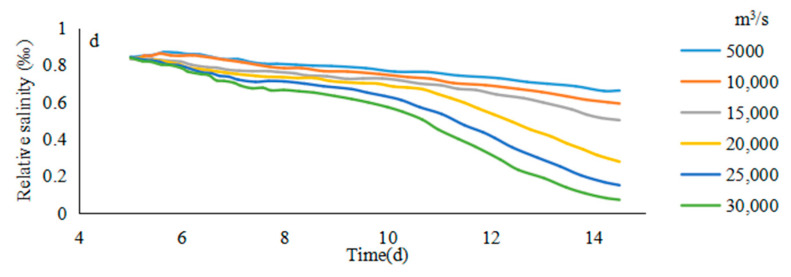
Relative salinity of the saltwater group in different river discharges and routes: (**a**) b1, (**b**) b2, (**c**) b3, (**d**) b4.

Under different flow condition, the salinity of the saltwater group on different paths at 0:00 on the sixth day was the initial salinity. The average relative salinity of the tidal cycle of the saltwater group core could be calculated over time based on the results of the numerical simulation (Figure 11), which was similar to the results of the previous absolute concentration analysis. The position of the saltwater group core moved down from each path, the decay of the relative salinity of the saltwater group accelerated significantly.

**Figure 12:**
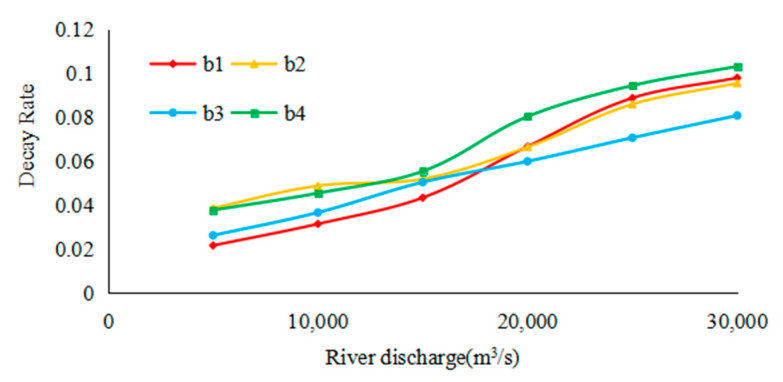
Relative salinity of the saltwater group in different river discharges and routes.

Further study on the decay rate of the saltwater group (Figure 12). The decay rate in different paths of the saltwater group was basically similar to the changing trend of river discharge. With the increase of river discharge, the decay rate is in the S-shaped trend of “fast-slow-fast”, which was closely related to the change of residence time of saltwater core in the mouth with the change of flow rate. The result accords with the study by Xu et al. [18].

### 3.4. Influences of the River Discharge on the Three Reservoirs

In order to better study the influence of saltwater spillover on the northern shore of the Yangtze River estuary on drinking water and industrial and agricultural production along the coast, calculation results of model B under different flow conditions. Extract from the three major reservoirs of the Yangtze River estuary (Chenghang Reservoir, Dongfengxisha Reservoir and Qingcaosha Reservoir), get the average daily salinity changes within half months, and understand the influence of flow rate on the salinity value and the changing process of the salinity value at water intake.

**Figure 13:**
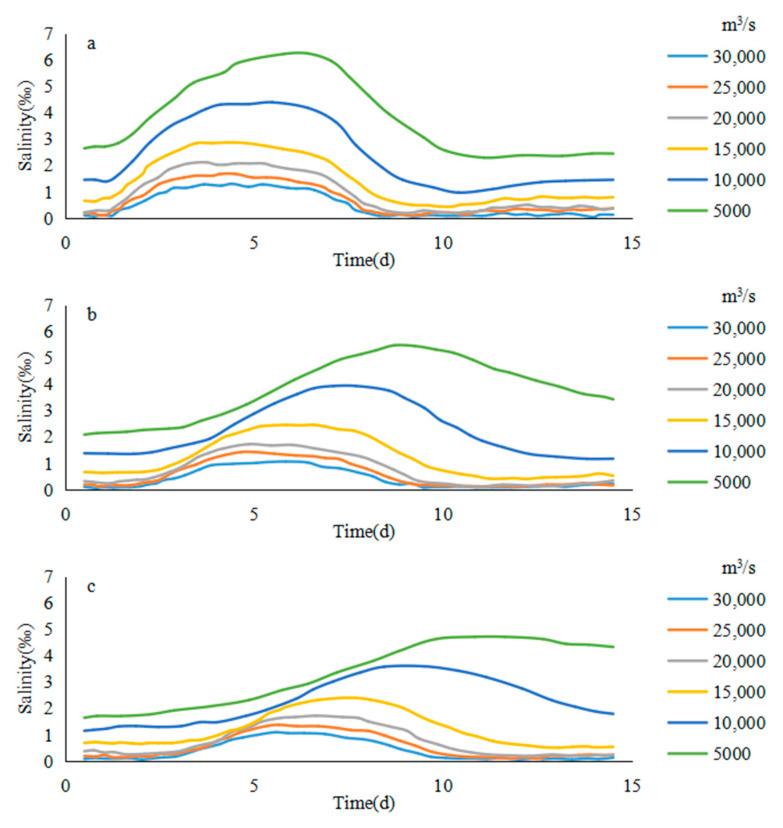
The average salinity change of reservoirs in different river discharges: (**a**) b1, (**b**) b2, (**c**) b3.

According to the daily average salinity change of the intake of Dongfengxisha Reservoir (Figure 13), it can be found that since Dongfengxisha Reservoir was located close to the NB of the spillover entrance, the spillover response during the rising tide was very fast. During the tide period (day 2–5), it remained at a high level for half a month. The flow and salinity were negatively correlated. When the flow was 5000 m^3^/s, the maximum salinity reached 7‰ in half a month. When the flow was 30,000 m^3^/s, the maximum salinity was about 2‰.

Since the water intake of Chenhang Reservoir is located about 30 km downstream of the water intake of the Dongfengxisha Reservoir, the maximum half-month salinity appeared later than the salinity peak of Dongfengxisha water intake. The maximum appeared at the end of low tide, middle tide and the spring tide. The higher the flow was, the earlier the maximum salinity appeared. This is consistent with the conclusion that the downward velocity of the saltwater group core and the flow are positively correlated in the previous section (when the flow is 5000 m^3^/s, intertidal may occur, resulting in an abnormal decrease in salinity).

The water intake of Qingcaosha Reservoir is about 10 km downstream of the water intake of Chenhang Reservoir, the maximum salinity within half a month was slightly later than Chenhang Reservoir. The overall salinity was lower than the corresponding salinity of the first two reservoirs.

In addition, the time when the maximum salinity of the water intake of the Chenghang Reservoir and the Qingcaosha Reservoir appeared was actually the time when the core of the saltwater group moved down the water intake of the two reservoirs (Table 5). Therefore, according to Equation (6) of the relationship between the position and time of the saltwater group on each path proposed in the previous section, the time when the maximum tide average salinity appeared can be estimated.

## 4. Discussion

### 4.1. Tidal Distributions

Tide-induced mixing mainly includes two aspects. On the one hand is the turbulent mixing caused by the frictional force of the river channel on the water during the period when the tide flows in the river channel. The second aspect is the interaction of tidal waves and rivers, which produces large-scale flows, including discrete shear flows, and the “pumping” and “blocking” effects of tidal waves. Under different tidal conditions, the fluctuation range of the saltwater group core within a tidal cycle increased with the tidal dynamics. With the increase of tide dynamics, the average downward velocity of the tide of the saltwater group core did not change much within the entrance, but it slowed down significantly near the entrance.

**Figure 14:**
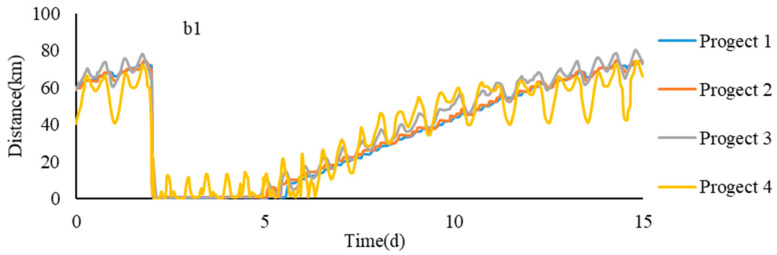
Average location of the saltwater group in different tidal intensity.

The model boundary hydrodynamic condition flow rate was 15,000 m^3^/s. We increased the tidal amplitude of the hydrodynamic boundary of the open sea by a certain proportion coefficient (see Table 6), kept the other conditions unchanged and performed simulation calculations to study the effects of different tidal intensities on the saltwater group. In this article, only path b1 was taken as an example. The position of the saltwater group changed under different tidal current conditions, as shown in Figure 14.

During the third to sixth day (during the period of the NB spillover tide), the core of the saltwater group oscillated near the original point, and there was no obvious downward movement. The stronger the tide is, the greater the amplitude of the oscillation. With the weakening of the NB spillover, the saltwater group started to move downward on the sixth day. When the tide was weak, there was almost no fluctuation during the downward movement of the saltwater group. Under the advection of the runoff, the core of the saltwater group moved basically at a uniform speed and moved downstream. As the tide power increased, the core of the saltwater group fluctuated significantly with the flood and ebb during a tide cycle. The stronger the tidal power, the larger the range of fluctuation.

### 4.2. Wind Distributions

Wind mainly drives the flow of water by friction on the surface of the water, which has an impact on the material transport of the water. Wind often acts as the main source of energy for large lakes, oceans and certain coastal areas, but in estuary areas, the role of wind may not be significant. For long and narrow estuaries, the tide and runoff generally have a major impact on the flow structure and material transport of the water. If the estuary is wider, the influence of wind power will increase relatively.

Due to the lack of a large amount of measured data, the effect of wind on the flow structure and material transport of the Yangtze River estuary is less studied. Wu Hui et al. [42] proved that northerly wind produced land-ward Ekman transport at the Yangtze River estuary with numerical simulations, and confirmed that this transport was helpful for spillover at the NB of the Yangtze River estuary. According to the measured data from Chongxi Hydrological Station, Li Lu [43] found that under the conditions of northerly winds, the salinity would increase abnormally during the low tide and mid tide. This further confirmed that wind has an important effect on the saltwater spillover at the Yangtze River Estuary, and based on this, the influence of wind on different transport was analyzed through the study of numerical simulation.

Based on model B, based on the upstream boundary flow of 1500 m^3^/s, the salinity changes of the saltwater group on the path b1 under the wind parameters of northerly 5 m/s, southerly 5 m/s, easterly 5 m/s and westerly 5 m/s were studied.

**Figure 15:**
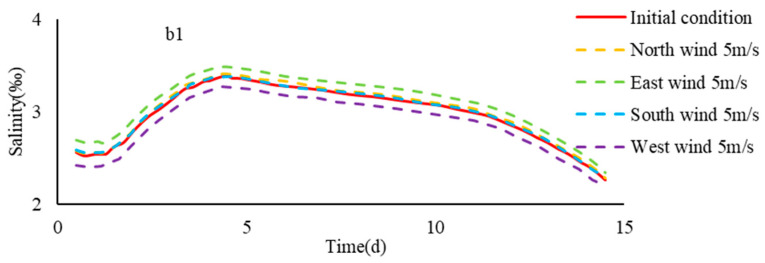
The average salinity of the saltwater group in different tidal intensity.

It can be seen from Figure 15 that different wind conditions had a certain influence on the activities and concentrations of NB spillover and spillover saltwater group, but its significance was smaller than that of runoff and current. Among them, the northerly and easterly could promote the NB spillover to a certain extent, and westerly could inhibit the NB spillover. The wind circulation from the north port to the south port formed by the north wind reduced the downward velocity of the saltwater group passing through the north port, while the downward velocity of the saltwater group passing through the south port increased.

### 4.3. Topography of the Area

Since the 21st century, the topography of the Yangtze River estuary has been changing rapidly under the influence of natural and artificial effects. In particular, the influence of the topographical changes in the NB on the spillover of the NB of the Yangtze River estuary has been the focus of attention. Based on the shoreline planned for the NB Reduction Project in 2004, Song Zekun [44] et al. used a mathematical model to study the changes of the tidal current characteristics of the NB, and the sediment and riverbed erosion and deposition of the NB below the planned shoreline. Wu Hui [42] studied the tide load, water level velocity and salinity changes in the NB, and analyzed the changes in the salinity of the SB water source point after the project.

During 2000–2008, the annual topographic change significantly weakened the inverted irrigation in NB, and the phenomenon of inverted irrigation will be further weakened after the implementation of the mid-narrowing scheme. The increase or decrease of the amount of back-irrigated saltwater caused by topographic change will make the salinity of the back-irrigated saltwater mass active in the southern branch increase or decrease significantly, but it has little effect on the activity law of the back-irrigated saltwater mass in SB [45].

## 5. Conclusions

In this paper, the transport of the saltwater group from the NB was studied. The author adopted the nesting model to better study the influence of spillover saltwater in the NB on the SB, selected different river discharge values to simulate salinity changes caused by different river flows. The main findings of this study are summarized as follows:

Taking b1 as an example, it can be seen from the moving position of the saltwater group core on path b1 under different runoff conditions that before the sixth day, due to the relatively stable spillover, no significant movement occurred. After the sixth day, the downward movement of the initial saltwater group became slowly. With the sharp decrease of the upstream spillover saltwater, the upstream saltwater supply was cut off by the drained runoff, and the movement of the saltwater group core accelerated gradually. At the end of the downward movement, the larger the runoff value, the further the downward movement distance reached by the saltwater group core, and converged to about 90 km gradually.

At different flow rates, the relationship between the average position and time of tide cycle of the saltwater group core of each water channel was consistent with the Gompertz model, and its parameters had a non-linear relationship with the flow rate. The fitting results of the model demonstrated that when the flow Q < 10,000 m^3^/s, the saltwater group core in each water channel could not move down to the entrance within half a month, and the continuous accumulation of the saltwater group in the entrance would cause serious saltwater pollution. As the flow increased, it took longer for the saltwater group core to reach the entrance, around 3–8 days. The average relative salinity decay rate of the saltwater group core in the entrance shows a S-shaped change that increased first and then decreased and then increased again along with the increase of the flow rate. This is closely related to the residence time of the saltwater group core in the entrance changing with the flow.

The semimonthly average tide salinity of the three major reservoirs along the Yangtze River Estuary appeared symmetrical hump. Under the same flow rate, the closer to upstream water intake, the bigger the hump curvature of the salinity change line; the closer to downstream water intake, the smaller the hump curvature of the salinity change line, which is related to the lower vertical gradient of the saltwater group. The hump of the average salinity process line at different tide levels decreased gradually with the increasing flow rate and the position of the maximum value advanced gradually with the increasing flow rate.

The fluctuation range of the saltwater group core increased with the tide current within a tidal cycle. With the increase of the tide dynamics, the average downward velocity of the tide of the saltwater group core did not change much within the entrance, but it slowed down significantly near the entrance. Different wind conditions had a certain influence on the activity and concentration of the NB spillover, but relatively less significance. The increase and decrease of the amount of the spillover saltwater caused by topographic changes will lead to marked increase or decrease of the salinity of spillover saltwater group in SB, whereas the impact on the activity rule of the saltwater group in SB will be little.

## Figures and Tables

**Table 1:** Model-observation data comparison statistics for tidal and salinity.

Quantity	Stations	Model A		Model B	
		Skill	Condition	Skill	Condition
Tidal current	Bm	0.93	Excellent	0.90	Excellent
	Stg	0.91	Excellent	0.89	Excellent
	Yl	0.94	Excellent	0.93	Excellent
	Hs	0.91	Excellent	0.88	Excellent
Salinity	Z4	0.70	Excellent	0.75	Excellent
	Z6	0.82	Excellent	0.84	Excellent
	Y4	0.78	Excellent	0.79	Excellent
	Y8	0.77	Excellent	0.75	Excellent

**Table 2:** Parameter calculation results of Gompertz model for b1 route.

River Discharge (m^3^/s)	*a*	*b*	*c*
5000	35.39	7.88	0.52
7500	47.46	5.74	0.462
10,000	60.41	3.69	0.42
12,500	68.68	2.97	0.42
15,000	74.64	2.65	0.44
17,500	80.62	2.44	0.46
20,000	83.45	2.42	0.51
22,500	86.11	2.5	0.56
25,000	88.4	2.68	0.63
27,500	89.81	2.89	0.68
30,000	91.62	3.15	0.78

**Table 3:** Parameter calculation results of Gompertz model for different routes.

Route	*a*	*b*	*c*
b1	32.3lnQ − 238.3	2 × 10^−8^Q^2^ − 8 × 10^−3^Q + 10.9	1 × 10^−9^Q^2^ − 3 × 10^−5^Q + 0.6
b2	37.5lnQ − 273	2.3 × 10^−8^Q^2^ − 9.6 × 10^−4^Q + 11.8	1.7 × 10^−9^Q^2^ − 5.4 × 10^−5^Q + 0.7
b3	33.2lnQ − 243.1	1.7 × 10^−8^Q^2^ − 8.5 × 10^−4^Q + 11.8	1.1 × 10^−9^Q^2^ − 3.6 × 10^−5^Q + 0.7
b4	31.5lnQ − 230.8	2.1 × 10^−8^Q^2^ − 8.4 × 10^−4^Q + 11.6	0.7 × 10^−9^Q^2^ − 0.9 × 10^−5^Q + 0.5

**Table 4:** Achieving the entrance time of the saltwater group in different river discharges and routes.

River Discharge (m^3^/s)	Route (Day)			
	b1	b2	b3	b4
12,500	8.099	7.361	8.275	7.982
15,000	5.939	5.975	6.290	5.973
17,500	4.813	4.527	4.755	4.617
20,000	3.984	3.598	3.818	3.733
22,500	3.478	3.000	3.315	3.178
25,000	3.141	2.749	3.174	2.930
27,500	2.945	2.639	3.214	2.815
30,000	2.763	2.585	3.175	2.722

**Table 5:** The time of maximum salinity in different reservoirs.

River Discharge (m^3^/s)	Dongfengxisha		Chenhang			Qingcaosha		
	Maximum Salinity (‰)	Time	Maximum Salinity (‰)	Time	Model Results	Maximum Salinity (‰)	Time	Model Results
5000	6.17	5.50	5.44	9.04	9.00	4.72	11.17	12.2
7500	5.27	5.21	4.66	8.25	8.50	4.18	10.25	10.54
10,000	4.38	4.92	3.89	7.46	8.0	3.64	9.33	9.30
12,500	3.28	4.79	2.93	6.42	6.42	2.75	7.46	7.82
15,000	2.95	4.61	2.59	5.94	6.45	2.46	7.17	7.45
17,500	2.62	4.42	2.25	5.46	5.78	2.17	6.88	7.05
20,000	2.35	4.42	2.00	5.42	5.39	1.94	6.60	6.81
22,500	1.88	4.42	1.57	5.17	4.81	1.52	6.29	6.49
25,000	1.66	4.42	1.37	4.96	4.56	1.33	6.25	6.43
27,500	1.47	4.42	1.21	4.96	4.58	1.18	6.23	6.41
30,000	1.29	4.42	1.05	4.96	4.87	1.02	6.21	6.38

**Table 6:** The projects of different tidal intensity.

Project	Coefficient
1	0.25
2	0.5
3	1
4	1.5
